# Regulation of MMP-3 expression and secretion by the chemokine eotaxin-1 in human chondrocytes

**DOI:** 10.1186/1423-0127-18-86

**Published:** 2011-11-25

**Authors:** Pin-Zhir Chao, Ming-Shium Hsieh, Chao-Wen Cheng, Yung-Feng Lin, Chien-Ho Chen

**Affiliations:** 1 Graduate Institute of Clinical Medicine, Taipei Medical University, Taipei, Taiwan; 2 Department of Otolaryngology, Taipei Medical University Shuang-Ho Hospital, New Taipei, Taiwan; 3 Department of Orthopedics, En Chu Kong Hospital, New Taipei, Taiwan; 4 School of Medical Laboratory Science and Biotechnology, Taipei Medical University, Taipei, Taiwan

**Keywords:** osteoarthritis, chemokine, cartilage degradation, chondrocyte, MMP-3, eotaxin-1

## Abstract

**Background:**

Osteoarthritis (OA) is characterized by the degradation of articular cartilage, marked by the breakdown of matrix proteins. Studies demonstrated the involvement of chemokines in this process, and some may potentially serve as diagnostic markers and therapeutic targets; however, the underlying signal transductions are not well understood.

**Methods:**

We investigated the effects of the CC chemokine eotaxin-1 (CCL11) on the matrix metalloproteinase (MMP) expression and secretion in the human chondrocyte cell line SW1353 and primary chondrocytes.

**Results:**

Eotaxin-1 significantly induced MMP-3 mRNA expression in a dose-dependent manner. Inhibitors of extracellular signal-regulated kinase (ERK) and p38 kinase were able to repress eotaxin-1-induced MMP-3 expression. On the contrary, Rp-adenosine-3',5'-cyclic monophosphorothioate (Rp-cAMPs), a competitive cAMP antagonist for cAMP receptors, and H-89, a protein kinase A (PKA) inhibitor, markedly enhanced eotaxin-1-induced MMP-3 expression. These results suggest that MMP-3 expression is specifically mediated by the G protein-coupled eotaxin-1 receptor activities. Interestingly, little amount of MMP-3 protein was detected in the cell lysates of eotaxin-1-treated SW1353 cells, and most of MMP-3 protein was in the culture media. Furthermore we found that the eotaxin-1-dependent MMP-3 protein secretion was regulated by phospholipase C (PLC)-protein kinase C (PKC) cascade and c-Jun N-terminal kinase (JNK)/mitogen-activated protein (MAP) kinase pathways. These data indicate a specific regulation of MMP-3 secretion also by eotaxin-1 receptor activities.

**Conclusions:**

Eotaxin-1 not only induces MMP-3 gene expression but also promotes MMP-3 protein secretion through G protein-coupled eotaxin-1 receptor activities. Chemokines, such as eotaxin-1, could be a potential candidate in the diagnosis and treatment of arthritis.

## Background

Osteoarthritis (OA) is a chronic degenerative joint disease characterized by degradation of articular cartilage and inflammation of the synovium [1,2]. Cartilage degradation is mediated by matrix metalloproteinases (MMPs), such as MMP-3 (stromelysin 1), which specifically cleave matrix proteins [3,4]. Chondrocytes, the only cells found in cartilage, can produce interleukin (IL)-1β that induces the expression of MMPs, aggrecanases, and other catabolic proteins [5,6]. Chondrocytes in OA cartilage may continuously be exposed to cytokines, chemokines and other catabolic factors at high local concentrations; however, the underlying effects and mechanisms are not well understood.

Chemokines are a family of small heparin binding cytokines that are primarily involved in the recruitment of leukocytes to the site of inflammation. Studies revealed roles of chemokines and catabolic cytokines in the inflammatory pathogenesis of OA [7,8]. Referring to the juxtaposition of cysteine residues in the protein's amino terminus, four subfamilies can be distinguished as C, CC, CXC, and CX3C [9]. In arthritic synovial tissue, IL-1β induces the production of the CC chemokines, such as monocyte chemoattractant protein 1 (MCP-1) and regulated upon activation of normal T cell expression and secretion (RANTES), and promotes inflammation [10,11]. It was also shown that chondrocytes respond to MCP-1 and RANTES by releasing MMP-3 and N-acetyl-β-D-glucosaminidase, thus contributing to cartilage matrix degradation [12]. Previously we demonstrated that MCP-1, RANTES and another chemokine, eotaxin-1 (CCL11), were overproduced in OA joints [13]. The plasma concentrations of these chemokines were higher in OA patients than in normal humans. The production of eotaxin-1 not only induces expression of its own receptors, CCR3 and CCR5, on the cell surface of chondrosarcomas, but also markedly increases the expression of MMP-3 mRNA in chondrocytes. Recent study also demonstrated elevated level of eotaxin-1 in the cells of rheumatoid arthritis (RA) patients before disease onset [14].

Eotaxin-1 was first isolated from lung lavage fluid of sensitized guinea pigs following allergen exposure [15]. The effects of eotaxin-1 are mediated by its binding to G-protein-coupled CC chemokine receptors (CCRs) [16,17]. Biochemical routes initiated by *Gα *subunit may activate the main secondary message signal, adenylyl cyclase-cAMP (AC-cAMP)-protein kinase A (PKA) pathway, and subsequently activate mitogen-activated protein (MAP) kinase pathway [18,19]. Activated MAP kinase translocates to the nucleus and phosphorylates transcription factors, thereby regulating gene expression [20,21]. On the other hand, the activated *G*βγ subunits may directly regulate phospholipase C (PLC)-protein kinase C (PKC) pathway [18]. The effect of G protein activation is mediated by both the AC-PKA and PLC-PKC cascades [22].

PLC is a key point of the pathway that regulates protein secretion. PLC has two major types including phosphatidylinositol specific phospholipase C (PI-PLC), and Phosphatidylcholine specific phospholipase C (PC-PLC). PI-PLC digests glycosyl-phosphatidylinositol-anchored protein on the pancreatic zymogen granule membrane to release the protein [23]. Acetylcholine activates insulin granules in pancreatic β-cells through PC-PLC pathway [24]. Furthermore, the effects on aldosterone secretion are initiated by an increase in Ca^2+ ^influx through hormone-operated Ca^2+ ^channels and G-protein- and PLC-dependent hydrolysis of phosphoinositides, leading to the generation of inositol 1,4,5 triphosphate (IP3) and diacylglycerol (DAG) that induces intracellular Ca^2+ ^release and PKC activation [25]. Ca^2+ ^influx and activation of PKC have been known for many years to be key signals of granule exocytosis and protein secretion. MMP-2 secretion from human ciliary muscle cells is regulated by PKC-dependent pathway [26]. PKC also stimulates the release of MMP-9 and tissue inhibitor of MMP1 in human decidual cells [27].

Mitogen-activated protein (MAP) kinase pathways regulate cell growth, differentiation, gene expression, protein synthesis and secretion. Three MAP kinase pathways have been studied in detail: extracellular signal-regulated kinase (ERK) 1/2, c-Jun N-terminal kinase (JNK), and p38 pathways. ERK 1/2 pathway is activated by growth factors, G-protein coupled receptors and phorbol esters, while the JNK and p38 MAP kinase pathways respond to environmental factors and inflammatory cytokines [28]. MAP kinases are involved in MMP mRNA production, and activated via different pathways with different inducers in different tissues [29]. IL-1 induces collagenase 3 (MMP-13) mRNA expression through p38 and JNK pathways in chondrocytes [30]; however, in osteoblastic cells, MMP-13 mRNA expression is activated via ERK pathway [31].

In our previous study, we found that eotaxin-1 at a high concentration induces MMP-3 mRNA production in the chondrocytes. We now show that eotaxin-1-induced MMP-3 expression is through cAMP/PKA and MAP kinase pathways. Eotaxin-1 at a low concentration is able to promote the MMP-3 release into the culture media. The induction of MMP-3 secretion by eotaxin-1 is regulated by PLC/PKC and MAP kinase pathways.

## Materials and methods

### Materials

Eotaxin-1 and IL-1β were purchased from R&D systems (Minneapolis, MN, USA). Inhibitors to ERK (PD98059), MAPK-ERK-kinase (MEK) (U0126), p38 (SB203580), JNK (SP600125), PI-PLC (U73122), PKA (KT5720), calcium (BAPTA-AM), and PKC (chelery chloride) were purchased from Tocris Bioscience (Bristol, UK). Inhibitors to AC (2',5'-dideoxyadenosine), PKA (H-89) and cAMP (Rp-cAMP) were purchased from Biomol International (Plymouth Meeting, PA, USA). Polyclonal antibody against MMP-3 was purchased from Oncogene Science (Cambridge, MA, USA), and antibody of glyceraldehyde 3-phosphate dehydrogenase (GAPDH) was from Zymed Laboratories (S. San Francisco, CA, USA). Materials from human subjects were obtained and processed under the regulation of TMU-Joint Institutional Review Board.

### Cell culture

Human SW1353 chondrosarcoma cells were purchased from ATCC (Manassas, VA, USA). Cells were seeded at a high density in Dulbecco's modified Eagle's medium (DMEM) containing 10% fetal bovine serum (FBS) (Gibco BRL), 100 U/ml penicillin and streptomycin, and incubated with 5% CO_2 _at 37°C. Osteoarthritis knee cartilage was obtained from patients undergoing total joint replacement surgery, and primary chondrocytes were prepared as described previously [32,33]. Cartilage slices were cut into pieces (2~3 mm^3^), and chondrocytes were released from articular cartilage by sequential enzymatic digestion with 1 mg/ml hyaluronidase (Sigma Chemical, St. Louis, MO, USA) for 15 min, 0.25% pronase (Sigma Chemical, St. Louis, MO, USA) for 30 min, then 2 mg/ml type II collagenase (Sigma) for 12 h in DMEM containing antibiotics (100 U/ml penicillin, 100 U/ml streptomycin, and 2.5 mg/ml amphotericin B) at 37°C. After filtration through a 100-meshnylon mesh and centrifugation, chondrocyte residues were washed and seeded at a high density in DMEM supplemented with 10% FBS and antibiotics, and incubated with 5% CO_2 _at 37°C.

### Reverse-Transcription Polymerase Chain Reaction (RT-PCR)

Total RNA was isolated from cultured cells, and RT-PCR was performed as described previously [13]. In brief, complementary DNA was synthesized in a 25-μl reaction mixture containing 5 μg of total RNA, 2.5 mM of each dNTP, 1 mM of random hexamer primers, and 10 U of M-MLV reverse transcriptase (Epicentre, Madison, WI), by incubation at 37°C for 90 min. The resulting cDNA (2 μl) was subjected to PCR using Taq DNA polymerase (Epicentre) and specific primers for MMP-3 and GAPDH. MMP-3 forward primer: 5'-CCTCTGATGGCCCAGAATTGA-3'; reverse primer: 5'-GAAATTGGCCACTCCCTGGGT-3' and GAPDH forward primer: 5'-CCACCCCATGGCAAATTCCATGGCA-3'; reverse primer 5'-TCTAGACGGCAGGTCAGGTCCACC-3'. For MMP-3, the PCR protocol was 35 cycles at 94°C for 1 min, 56°C for 1 min, and 72°C for 1 min. In each experiment, amplification of cDNA for the housekeeping gene, GAPDH, was used as an internal standard. PCR products were analyzed on 1.5% agarose gels.

### Western blot analysis and determination of MMP-3

Proteins were separated in SDS-PAGE according to standard protocol and transferred onto PVDF-nylon membranes (Millipore). The membrane was blocked with 5% non-fat milk in TBST (10 mM Tris-HCl, pH7.5, 150 mM NaCl, and 0.1% Tween-20) at room temperature for 1 h. After a brief wash, the membrane was incubated with primary antibody diluted in TBST for 60-90 min. The membrane was then washed 3 times with TBST and probed with horseradish peroxidase-conjugated secondary antibody (1:3000, Santa Cruz Biotechnology, Santa Cruz, CA) for 30-60 min. After extensive washes, specific signals were visualized by an enhanced chemiluminescence (ECL) system (Pierce, Rockford, IL) according to the manufacture's instruction. Western bands were digitalized and quantified by UN-SCAN-IT gel 6.1 software (Silk Scientific Inc., Orem, UT, USA).

### IP3 detection

Cells were suspended in the phosphate-buffered saline (PBS), and were incubated with 0.2 volume of ice-cold 20% trichloroacetic acid on ice for 20 min. The protein sediment was precipitated by centrifugation at 2000 × *g *for 15 min at 4°C, and the supernatant was adjusted with ice-cold 10 M KOH to pH 7.5. The KClO_4 _sediment was removed by centrifugation at 2000 × *g *for 15 min at 4°C. The Ins(1,4,5)P_3 _level in supernatant was determined using Inositol-1,4,5-Trisphosphate [^3^H] Radioreceptor Assay Kit (Amersham Biosciences).

### Statistical analysis

The mean and standard deviation (SD) were used to illustrate the results from at least three data sets of each experiment. Statistical significance (*p *< 0.05) was assessed using Student's test or one-way analysis of variance, followed by a post hoc analysis using Dunnett's test when appropriate.

## Results

### Eotaxin-1 induces MMP-3 gene expression and protein secretion in human chondrocytes

**Figure 1:**
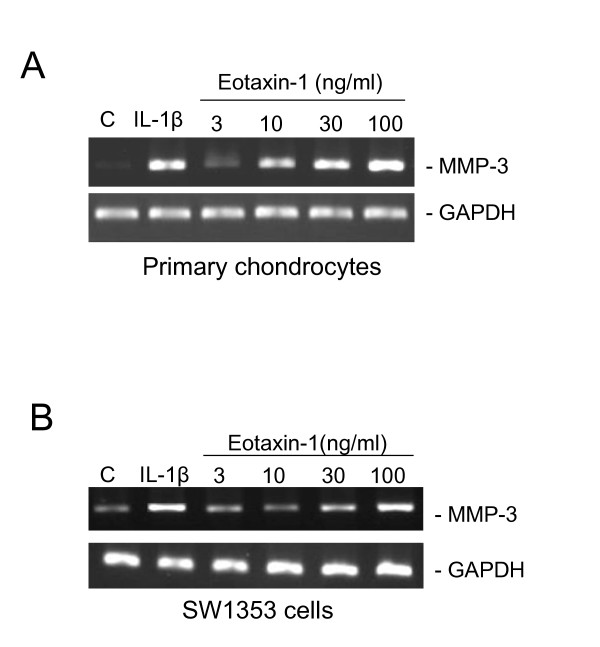
**Effect of eotaxin-1 on the MMP-3 gene expression in human chondrocytes**. Primary human chondrocytes (A) and SW1353 chondrosarcoma cells (B) were incubated with various concentrations of Eotaxin-1 or IL-1β (1 ng/ml) for 6 h, and MMP-3 mRNA expression was detected by RT-PCR. These experiments were performed three times.

In our previous study, we found that eotaxin-1 is overexpressed in OA patients [13]. Eotaxin-1 induces MMP-3 mRNA expression in human chondrocytes. Similarly in the present study, we demonstrated that MMP-3 expression in SW1353 chondrosarcoma cells and primary chondrocytes was obviously induced by eotaxin-1 at 30 and 10 ng/ml, respectively (Figure 1A and 1B). It is notable that treatment with eotaxin-1 alone was able to induce MMP-3 expression in both primary chondrocytes and a chondrosarcoma cell line. However, we treated cells with IL-1β in addition to eotaxin-1 in most of further experiments to magnify the overall effects.

**Figure 2:**
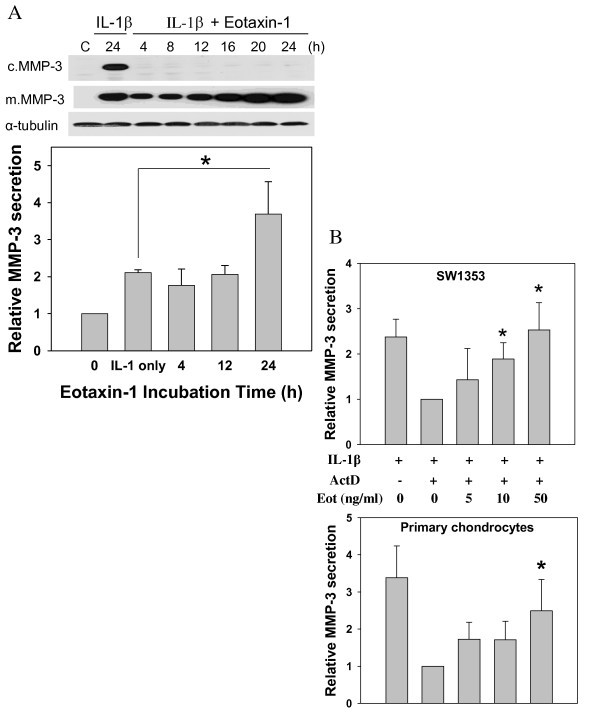
**Effect of eotaxin-1 on the MMP-3 protein secretion in human chondrocytes**. Human SW1353 chondrosarcoma cells and primary chondrocytes were cultured and treated in different conditions. MMP-3 protein levels were analyzed by Western blotting, and quantified. All values were normalized to the one without eotaxin-1. (A) SW1353 cells were incubated with 1 ng/ml IL-1β and 100 ng/ml eotaxin-1 for various time periods or only IL-1β for 24 h, and MMP-3 protein expressed in cells (c.MMP-3) and culture media (m.MMP-3) were analyzed and quantified. (B) SW1353 or primary cells were incubated with IL-1β (1 ng/ml) for 4 h followed by actinomycin D (5 μg/ml) for 1 h. The medium was then refreshed, and the cells were treated with various concentrations of eotaxin-1 for 2 h. MMP-3 protein in culture medium was determined. The values are the mean of at least three assays. * *p *< 0.05.

In order to check the eotaxin-1-induced MMP-3 protein levels in chondrosarcoma cells, we performed Western blotting using cell lysates and culture media. With only IL-1β treatment (1 ng/ml) for 24 h, MMP-3 protein was present in both the cytosol and culture media. Surprisingly, after treating the cells with 100 ng/ml eotaxin-1 along with IL-1β, MMP-3 protein levels were not detected in cell lysates at the time points from 4 h to 24 h, and only found in the culture media (Figure 2A). The levels of MMP-3 protein in culture media increased with time.

To clarify the effect of eotaxin-1 on MMP-3 secretion, we used actinomycin D (ActD) to eliminate the effects from MMP-3 expression. ActD is an inhibitor of transcription, and has been used, at concentrations ranging from 1 to 10 μg/ml, to inhibit gene expression in human chondrocytes [34,35]. In the presence of ActD (5 μg/ml or 4 μM), IL-1β-induced MMP-3 protein level in culture media was reduced, especially in primary cell cultures, suggesting efficient suppression of MMP-3 gene by ActD (Figure 2B). Indeed, eotaxin-1 at moderate concentrations still significantly promoted the MMP-3 protein level in culture media after the transcription was inhibited. Since the inhibition of transcription of MMP-3 did not block the effect of eotaxin-1 on promoting MMP-3 levels in culture media, the phenomena may be attributed to the eotaxin-1-enhanced secretion of MMP-3 protein. It was noted that primary cells were less responsive to eotaxin-1 than SW1353. Perhaps the primary chondrocytes from OA patients were customized to high eotaxin-1 concentrations.

It is plausible that eotaxin-1 not only induced MMP-3 gene expression but also promoted the protein secretion into culture media from human chondrocytes.

### RANTES and MCP-1 induce MMP-3 gene expression but not protein secretion

**Figure 3:**
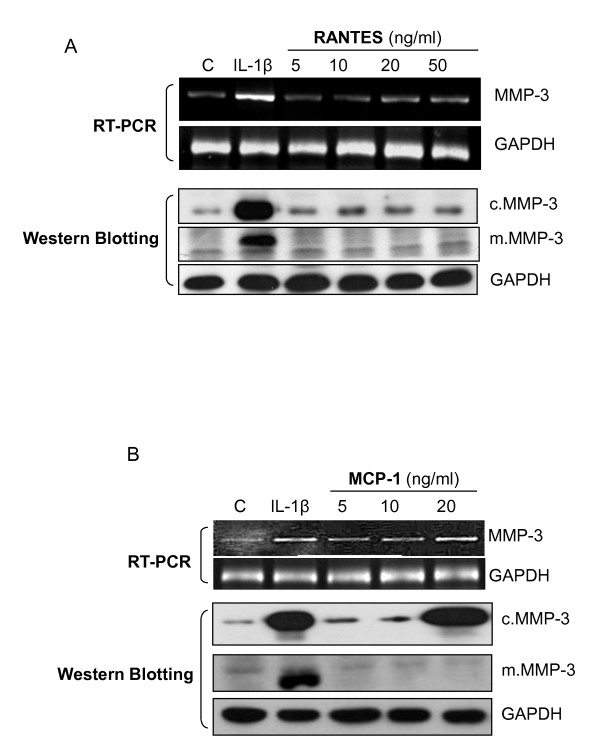
**Effects of other CC chemokines on the MMP-3 gene expression and protein secretion in human chondrosarcoma cells**. SW1353 cells were incubated with 1 ng/ml IL-1β, or various concentrations of RANTES (A) or MCP-1 (B) for 24 h. MMP-3 mRNA levels were analyzed by RT-PCR, and the protein expressed in cells (c.MMP-3) and secreted in media (m.MMP-3) were assayed by Western blotting. These experiments were performed three times.

Our earlier results also indicated high plasma concentrations of the other two chemokines, RANTES and MCP-1α in OA patients [13]. Therefore we checked their effects on MMP-3 mRNA expression, and protein levels in cells and media. As shown in Figure 3, both RANTES and MCP-1 at moderate concentrations increased the level of MMP-3 mRNA (Figure 3A and 3B, upper panels). Similar to eotaxin-1 and consistent with the previous report, RANTES and MCP-1 are involved in MMP-3 gene regulation. However, greater protein levels of MMP-3 were found in cell lysates than in culture media in both experiments (Figure 3A and 3B, lower panels), suggesting that RANTES and MCP-1 are not involved in regulation of MMP-3 secretion.

### MAP kinases are involved in eotaxin-1-induced MMP-3 gene expression and protein secretion

**Figure 4:**
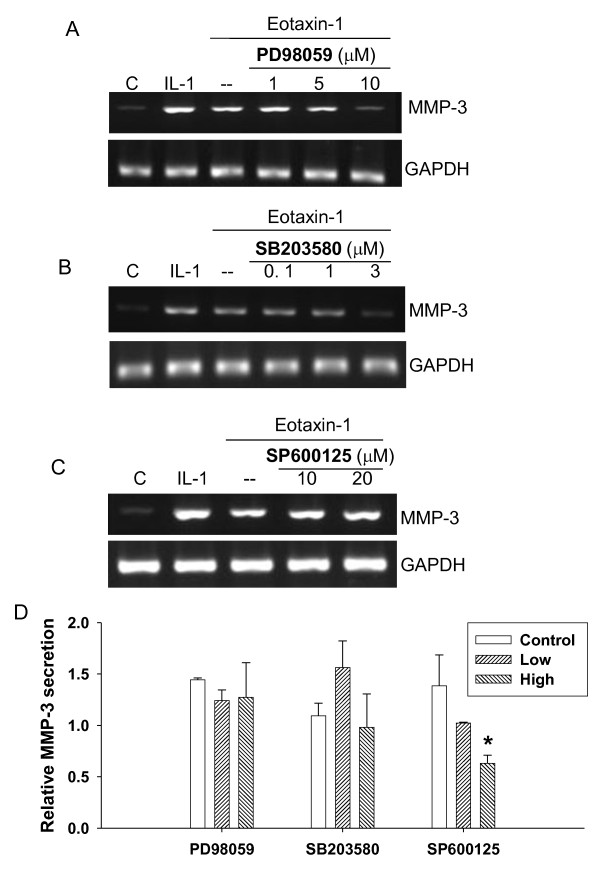
**Involvement of MAP kinases in eotaxin-1-induced MMP-3 gene expression and protein secretion in human chondrosarcoma cells**. SW1353 cell were pretreated for 1 h with inhibitors as follows: ERK inhibitor PD98059 at 1, 5 and 10 μM (A), p38 inhibitor SB203580 at 0.1, 1 and 3 μM (B), and JNK inhibitor SP600125 at 10 and 20 μM (C). The cells were then treated with eotaxin-1 (100 ng/ml) or IL-1β (1 ng/ml) for 6 h. MMP-3 mRNA expression was assayed by RT-PCR. (D) MMP-3 protein secretion of the cells treated with the inhibitors was also analyzed by Western blotting and quantified. Concentrations of the inhibitors used from low to high: 10 and 20 μM PD98059, 5 and 10 μM SB203580, 2.5 and 5 μM SP600125. These experiments were performed at least three times. The values in the plot were the mean of at least three assays.

To investigate the pathways that involve eotaxin-1 and MMP-3, we used inhibitors of ERK, p38, and JNK MAP kinases. The eotaxin-1-induced mRNA levels of MMP-3 were apparently decreased by the inhibitors of ERK (PD98059) at 10 μM and p38 (SB203580) at 3 μM, but not JNK (SP600125) at 20 μM (Figure 4A, B and 4C). This suggests the involvement of both ERK and p38 in the regulation of eotaxin-1 signaling through MMP-3 expression in chondrocytes.

The effects of these inhibitors on MMP-3 secretion in the cells were then examined. The ERK and P38 inhibitor concentrations that are higher than those effective in reducing MMP-3 gene expression did not inhibit eotaxin-1-induced MMP-3 protein secretion (Figure 4D). In contrast, a low concentration of JNK inhibitor significantly reduced MMP-3 protein secretion which was induced by eotaxin-1 in a dose-dependent manner. This indicates a role for JNK in the pathway of eotaxin-1-induced MMP-3 protein secretion in chondrocytes.

### AC/PKA is inhibitory in eotaxin-1-induced MMP-3 gene expression

**Figure 5:**
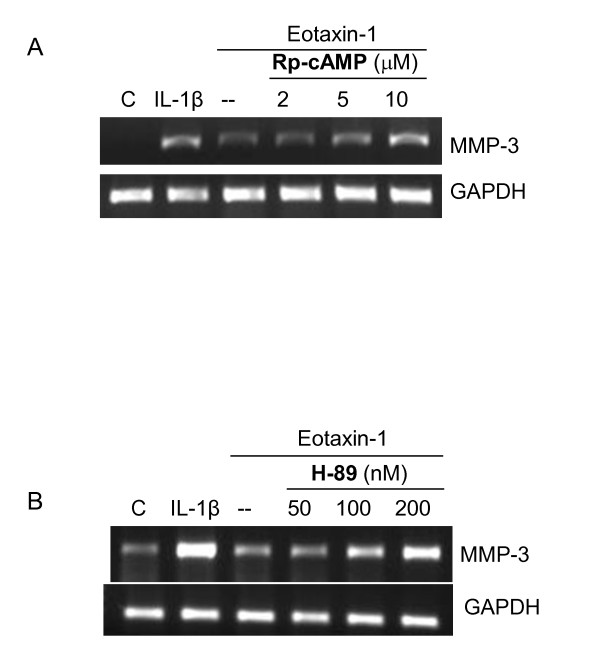
**Involvement of AC/PKA in eotaxin-1-induced MMP-3 gene expression in human chondrosarcoma cells**. SW1353 cell were pretreated with various concentrations of cAMP inhibitor Rp-cAMP (A) or PKA inhibitor H-89 (B) for 1 h, and then treated with eotaxin-1(100 ng/ml) or IL-1β (1 ng/ml) for 6 h. MMP-3 mRNA expression was assayed by RT-PCR. These experiments were performed three times.

Rp-cAMP inhibits cAMP on the activation of downstream proteins, such as PKA. Chondrosarcoma cells were pretreated with Rp-cAMP prior to the treatment with eotaxin-1. Interestingly Rp-cAMP increased the level of eotaxin-1-induced MMP-3 mRNA at moderate concentrations (Figure 5A). Consistent with the finding, PKA inhibitor also increased the level of MMP-3 mRNA at low concentrations. These results indicate that AC/PKA is inhibitory in eotaxin-1 signal transduction by down-regulating MMP-3 expression. Eotaxin-1 may indeed activate MAP kinases by inhibiting AC/PKA activities.

### PI-PLC is involved in eotaxin-1-induced MMP-3 protein secretion

**Figure 6:**
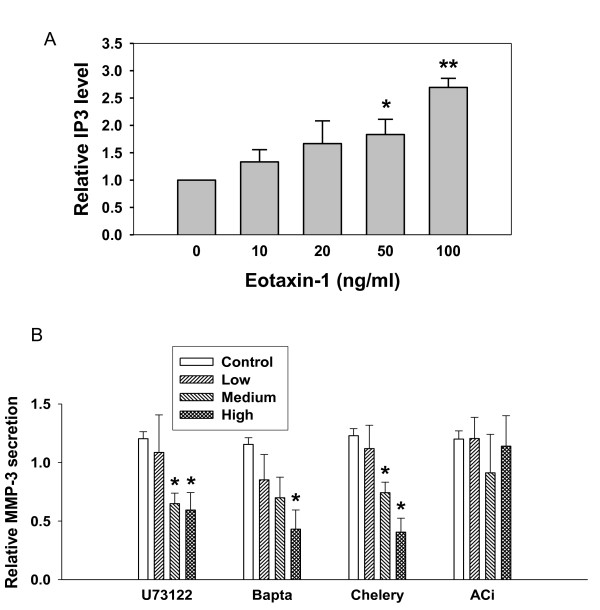
**Involvement of PI-PLC in eotaxin-1-induced MMP-3 secretion in human chondrosarcoma cells**. (A) SW1353 cells were treated with various concentrations of eotaxin-1 for 30 min, and determined IP_3 _level by IP_3 _[^3^H] radioreceptor assay. (B) SW1353 cell were treated for 4 h with IL-1β (1 ng/ml) followed by 1 h with actinomycin D (5 μg/ml) and various inhibitors as follows: PI-PLC inhibitor U73122 (5, 10 or 20 μM), calcium inhibitor Bapta-AM (Bapta; 5, 10 or 20 μM), PKC inhibitor chelery chloride (Chelery; 0.5, 1 or 2 μM), or adenylate cyclase inhibitor 2',5'-dideoxyadenosine (ACi; 100, 250 and 500 μM). The cells were then treated with eotaxin-1 (100 ng/ml) for 4 h. MMP-3 protein secreted in culture media was determined by Western blotting and quantified. The values in the plots were the mean of at least three assays. * *p *< 0.05.

IP3 is a catalytic product of PLC, and IP3 level indicates the activity of PI-PLC pathways. As shown in Figure 6A, IP3 levels were increased by eotaxin-1 in a dose-dependent manner. Eotaxin-1 may activate phospholipase C, and increase the production of IP3 at a concentration lower than 100 ng/ml. Cells were further tested by treating with inhibitors of PLC (U73122), calcium (BAPTA-AM), PKC (Chelery chloride), or adenylate cyclase (ACi) prior to the treatment with eotaxin-1 (10 ng/ml). The levels of secreted MMP-3 protein were decreased in a dose-dependent manner by inhibitors of PLC, calcium and PKC, but not adenylate cyclase (Figure 6B). These data indicate that both PLC/PKC pathway and the calcium influx may be involved in eotaxin-1-induced MMP-3 protein secretion.

## Discussion

**Figure 7:**
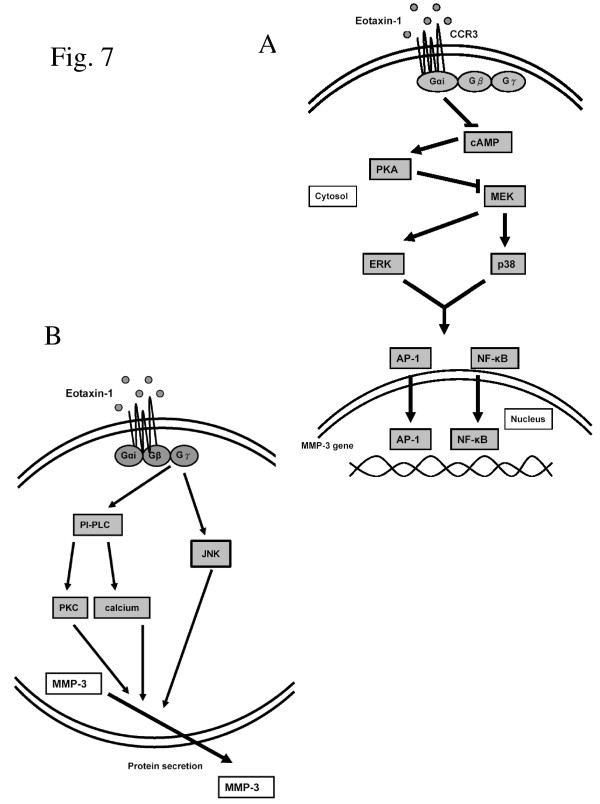
**Models of eotaxin-1 function on MMP-1 gene expression and protein secretion in human chondrocytes**. (A) Eotaxin-1 binds G protein-coupled CCRs, such as CCR3, and induces MMP-3 expression through cAMP/PKA inhibition and MAP kinase activation. (B) Eotaxin-1 at low concentrations is able to promote MMP-3 release from the cells by activating PLC/PKC and MAP kinase pathways.

Chondrocytes are major cells of cartilage in joints, and are implicated in the pathology of OA which is a multifactorial disease. One of the factors is imbalance of MMPs. In our previous study, MMP-3 is highly correlative with OA by increasing collagen degradation in the cartilage matrix [13]. In the plasma and synovial fluid of OA patient, two catabolic cytokines, IL-1β and TNF-α, and several chemokines including eotaxin-1 were highly expressed. The release of MMP-3 from chondrocytes and synoviocytes in response to the stimulations may play a major role in the progressive cartilage disruption in OA patients. In this study, the signal transduction pathways regulating MMP-3 gene expression and protein secretion in response to eotaxin-1 in human chondrocytes were investigated. The results demonstrated that the three examined chemokines (RANTES, MCP-1, and eotaxin-1) were able to induce the expression of MMP-3; however, only eotaxin-1 was able to promote the secretion of MMP-3 from the cells. Further experiments demonstrated that eotaxin-1 may inhibit cAMP/PKA, and activate ERK and p38 MAP kinases to induce MMP-3 expression (Figure 7A). Meanwhile eotaxin-1 signaling may also be mediated by PLC-PKC cascade, and JNK MAP kinase pathway to promote MMP-3 secretion (Figure 7B).

The eotaxin-1 receptor CCR3 expressed on SW1353 chondrosarcoma cells belongs to the family of G protein-coupled receptors. The effects of eotaxin-1 were sensitive to pertussis toxin. Eotaxin-1 stimulation results in a rapid decrease of cAMP levels indicating association of the eotaxin-1 receptors with *G*α_i _proteins. Addition of cAMP inhibitor (Rp-cAMP) enhanced the effects of eotaxin-1-induced transcription (Figure 5A). This finding supports that cAMP plays a central role in eotaxin-1-induced MMP-3 expression. A key target for cAMP is PKA. The PKA inhibitor also increased the effects of eotaxin-1 by inducing MMP-3 transcription in chondrosarcoma cells (Figure 5B). These results indicate that AC/PKA negatively modulates transcription of MMP-3 in chondrosarcoma cells.

MEK lies at the key point of a signaling network that controls cell proliferation, neoplastic transformation, and differentiation. Many of these effects are transmitted via the MAP kinase pathway. The inhibitors of ERK and p38 MAP kinases decreased the mRNA level of MMP-3 (Figure 4A and 4B). It implicates that these MAP kinases are involved in MMP-3 transcription induced by eotaxin-1. Similar effect by other chemokines in human articular chondrocytes was also reported recently [36]. The cross-talk of PKA and MAP kinase pathways was discussed in previous studies [19,37]. MAP kinases are regulated by cAMP/PKA pathway, and PKA also cross-talks with Raf-1, indicating that MAPK could control transcription through AP-1 and NF-*κ*B. These observations conclude direct relevance of eotaxin-1 to MMP-3 expression in osteoarthritis.

Interestingly, the JNK inhibitor, SP600125, did not inhibit eotaxin-1-induced MMP-3 expression at relatively high concentrations (Figure 4C). Similar effects of different stimuli on MAP kinase pathways to MMP expression in chondrocytes were also reported in recent studies. Leptin, produced by joint white adipose tissue, induced MMP-1 and MMP-13 expression in chondrocytes [38]. Inhibitors of ERK and p38 pathways significantly reduced those MMPs expression; however, JNK inhibition had no effect on leptin-induced MMP-13 expression. Mechanical stress-induced MMP-13 was down-regulated by p38 inhibitor SB203580 but not by the ERK inhibitor U0126, or the JNK inhibitor JNK inhibitor II in another report in another report [39]. The JNK seemed to distinguish itself from other MAP kinases in regulating MMP activities in chondrocytes. Indeed, our data suggested an important pathway for eotaxin-1 to stimulate MMP secretion via JNK MAP kinase (Figure 4D and 7B).

Since the *G*_i _protein is one of the subunits composed of eotaxin-1 receptor, CCR3, it is believed that *G*_i_-coupled receptors are primarily mediated by βγ-subunit complex to activate MAP kinase. One mechanism appears to be PI3K dependent. Signaling from PI3K to MAP kinase pathway requires a tyrosine kinase, indicating that the GPCR is involved. It is known that binding of eotaxin-1 to CCR3 activates not only *Gα*_*i *_subunit but also *G*βγ that potentially related to protein secretion [40]. PLC is the key molecule of regulating protein secretion pathways. Stimulation of chemokine receptors rapidly activates PI-specific PLC, which leads to IP3 formation and a transient rise in the concentration of intracellular free calcium. Our data show that inhibition of PLC by U73122 abolishes eotaxin-1-induced MMP-3 release (Figure 6B). This is evident that PI/PLC is involved in the regulation of MMP-3 secretion pathway induced by eotaxin-1. There were studies showing the involvement of PLC in gene regulation of MMP-3 in fibroblasts [41] and other MMPs in chondrosarcoma cells [42]. It is possible that PLC is also involved in the eotaxin-1-induced MMP-3 gene expression. Further experiments may be performed in future studies.

Activated PLC has been reported to stimulate IP3, calcium influx, and PKC in a number of cell types. The stimulation of neutrophils by receptor-binding ligands can activate PLC with the formation of IP3 which releases Ca^2+ ^from intracellular storage, and DAG which activates PKC [43]. Indeed our results show that eotaxin-1 stimulation resulted in a rapid increase of IP3 levels, and inhibition of calcium and PKC decreases the MMP-3 protein secretion induced by eotaxin-1 (Figure 6). The MMP-3 protein secretion induced by eotaxin-1 is, thereby, calcium dependent, and associated with *G*βγ proteins and PLC. Moreover, eotaxin-1-activated PLC not only induced intracellular calcium release but also activated PKC. Activation of PKC by eotaxin-1 suggests a potential role for PKC-induced MMPs in the mechanisms responsible for membrane rupture. Recent studies showed that activation of PKC is involved in the induction of MMP secretion by cytokines in smooth muscle cells [44]. Our data clearly show that PKC inhibitor significantly decreased the secretion of MMP-3 in a dose-dependent manner. PKC is, therefore, involved in eotaxin-1-induced MMP-3 secretion pathway.

## Conclusions

Human chondrocytes respond to the stimulation of eotaxin-1 by up-regulating MMP-3 expression and secretion, which may be mediated by *G*α_i _and *G*βγ subunits of *G*-coupled protein receptor, respectively. High concentrations of eotaxin-1 inactivate cAMP/PKA, and spark ERK and p38 MAP kinases to regulate MMP-3 transcription. Yet, at low concentrations, eotaxin-1 activates PI3K and JNK MAP kinase to stimulate secretion of MMP-3, which plays an important role in OA pathogenesis. Critically, eotaxin-1 not only induces MMP-3 transcription but also enhances MMP-3 secretion. Our results shed light on key roles of eotaxin-1 in cartilage destruction in OA, and suggest a potential diagnostic and therapeutic target for this disease.

## Abbreviations

AC: adenylyl cyclase; ActD: Actinomycin D; CCL11: CC chemokine eotaxin-1; CCR: CC chemokine receptors; DAG: diacylglycerol; DMEM: Dulbecco's modified Eagle's medium; ECL: enhanced chemiluminescence; ERK: extracellular signal-regulated kinase; FBS: fetal bovine serum; IL: interleukin; IP3: inositol 1,4,5 triphosphate; GAPDH: glyceraldehyde 3-phosphate dehydrogenase; JNK: c-Jun N-terminal kinase; MAP: mitogen-activated protein; MCP-1: monocyte chemoattractant protein 1; MEK: MAPK-ERK-kinase; MMP: matrix metalloproteinase; OA: osteoarthritis; PBS: phosphate-buffered saline; PC: phosphatidylcholine; PI: phosphatidylinositol; PKA: protein kinase A; PKC: protein kinase C; PLC: phospholipase C; RA: rheumatoid arthritis; RANTES: regulated upon activation of normal T cell expression and secretion; RT-PCR: reverse-transcription polymerase chain reaction;

## Competing interests

The authors declare that they have no competing interests.

## Authors' contributions

PZC designed and performed research. MSH performed research and wrote paper. CWC analyzed data. YFL analyzed data and wrote paper. CHC designed research and wrote paper.
